# Numerical Modeling Strategies in Flax Fiber-Reinforced Polypropylene Forming Processes

**DOI:** 10.3390/ma19153254

**Published:** 2026-08-01

**Authors:** Antonio Formisano, Ilaria Improta, Giuseppe Irace

**Affiliations:** 1 Department of Chemical, Materials and Production Engineering, University of Naples Federico II, P.le V. Tecchio 80, 80125 Napoli, Italy; giuseppe.irace@unina.it; 2 Institute of Polymers, Composites and Biomaterials, National Research Council, P.le E. Fermi 1, 80055 Portici, Italy; ilariaimprota@cnr.it

**Keywords:** incremental forming, stretch forming, polypropylene, flax fibers, finite element simulations, forming forces, elastic springback, failures

## Abstract

Natural fiber-reinforced thermoplastic composites have gained increasing attention due to the abundant availability of natural fibers, their ability to effectively reinforce polymer matrices, and the resulting partial biodegradability of the final material. This study presents numerical modeling strategies for investigating the manufacture of spherical caps made from polypropylene composites reinforced with woven flax fabrics. The investigation considers both cold incremental forming and stretch-forming processes, performed either with or without the support of a partial counter die. Building on the findings of a previous experimental study conducted by the authors on compression-molded laminates manufactured using untreated woven flax fabrics and without coupling agents and formed without localized heating, numerical predictions are compared with experimental results in terms of final geometry, forming forces, and failure mechanisms. The findings highlight the need for a thorough understanding of material behavior to fully exploit finite element analysis as a reliable predictive tool in the forming of these innovative lightweight composite structures.

## 1. Introduction

In recent years, the incorporation of natural fibers derived from seeds, stems, and roots as reinforcing elements in polymer composites has attracted considerable attention from both the scientific community and industry [1]. Their growing popularity stems from their ability to improve selected properties of conventional polymer matrices, potentially reduce the energy requirements associated with processing, and impart a degree of biodegradability to the resulting composites [2]. Furthermore, natural fibers offer a sustainable and economically attractive alternative to traditional synthetic reinforcements, such as glass and carbon fibers, owing to their renewable origin, low cost, biodegradability, and non-toxic nature [3].

Among natural reinforcements, flax and hemp fibers are recognized as the strongest and stiffest options available [4]. Flax is also one of the most extensively cultivated and readily available natural fibers in Europe, making it particularly attractive for composite applications. Owing to its low density, high specific stiffness, and excellent vibration-damping capability compared with conventional reinforcements such as glass and aramid fibers, flax has become a preferred material for the development and production of bio-based composite structures [5].

Thermoplastic polymers are widely employed as matrices for composite materials across numerous industrial sectors, including automotive [6], aerospace [7], and biomedical applications [8]. Compared with thermosetting resins, thermoplastics offer several advantages, such as shorter processing cycles, recyclability, and the ability to be reshaped and reprocessed when subjected to elevated temperatures [9,10]. Among thermoplastic materials, polypropylene (PP) is the second most extensively produced synthetic polymer worldwide. Its widespread use is attributed to its excellent combination of chemical resistance, wear resistance, favorable mechanical performance, ease of manufacturing, and low cost [11]. As a result, PP is commonly utilized in the production of automotive components, reusable containers, packaging systems, and laboratory equipment [12], as well as in advanced composite structures for aerospace, civil engineering, and transportation applications [13]. Furthermore, together with polyethylene and polyvinyl chloride, PP remains one of the most commonly adopted polymer matrices for natural fiber-reinforced composites, owing to its versatility, availability, and compatibility with a broad range of lignocellulosic reinforcements [14].

Among the advanced manufacturing technologies that have recently attracted growing attention for the processing of non-metallic materials, including thermoplastic polymers and composite laminates, incremental sheet forming (ISF) stands out as a particularly promising solution. In its most basic configuration, referred to as negative incremental forming (NIF), the process consists of the gradual deformation of a clamped sheet by means of a simple, non-dedicated forming tool that follows a predefined CNC-controlled toolpath to progressively generate the desired geometry [15]. The layer-by-layer nature of ISF makes it especially suitable to produce customized components and complex free-form geometries [16]. Consequently, ISF has found applications in a wide range of sectors, including aerospace [17], automotive [18], medical engineering [19,20], and other advanced manufacturing fields.

The application of ISF to thermoplastic materials has been extensively investigated [21,22,23], largely due to their widespread use in mass-production industries [24]; research efforts have demonstrated that the process can effectively replace conventional manufacturing routes based on repeated heating, forming, and cooling cycles [25,26]. In contrast, studies focusing on the ISF of composite materials are still relatively limited. Nevertheless, starting from the pioneering investigations reported in the literature [27,28] and progressing toward more recent developments involving glass fiber-reinforced [29] and carbon fiber-reinforced polymer composites [30], increasing evidence suggests that ISF represents a viable, sustainable, and economically attractive alternative for the manufacturing of composite structures [31].

Numerous studies have highlighted both the strengths and limitations of ISF. Compared with conventional sheet-forming technologies, ISF offers several advantages, including reduced tooling requirements, lower production costs, shorter setup times, enhanced formability, greater process flexibility, and a reduced environmental footprint. However, it is also characterized by relatively long manufacturing times and limited geometric accuracy, mainly due to the absence of dedicated counter dies [32,33,34,35]. In a previous study [36], the authors investigated and compared ISF and stretch-forming (SF) processes applied to compression-molded PP composites reinforced with woven flax fabrics, manufactured without fiber surface treatments or coupling agents. Spherical caps were produced at room temperature, both with and without the support of a partial counter die, and their mechanical performances were evaluated by penetration tests. The study highlighted the effectiveness of ISF, particularly when combined with a partial counter die, for producing the desired components with very low levels of forming force and energy consumption, further enhanced by the absence of any heating stage; moreover, the spherical caps exhibited improved mechanical performance compared to the undeformed laminates, making them suitable as stiffening elements for lightweight panels in a variety of industrial sectors.

Computational modeling techniques allow researchers to gain a deeper understanding of the mechanical response and forming behavior of composite materials; among these, the finite element method (FEM) is one of the most widely adopted numerical approaches for solving complex engineering and physical problems [37]. In recent years, multiscale and multiphysics modeling strategies have emerged as some of the most advanced approaches for the simulation of composite materials [38], and conventional FEM methodologies generally rely on either macroscale or microscale analyses, both requiring an extensive and complex mechanical characterization of the material. In macroscale analyses, the mechanical properties are assigned directly to each ply of the laminate stacking sequence, whereas microscale approaches typically employ homogenization techniques based on the definition of a representative volume element (RVE) to determine the effective material behavior [39,40]. An alternative strategy involves the adoption of micromechanical models, in which the constituent phases of the composite (including fibers, matrix, and cohesive interfaces) are explicitly represented within the numerical framework, allowing for a more detailed description of the underlying damage and failure mechanisms [41].

This study presents a FEM-based numerical framework for investigating the manufacture of the previously described spherical caps, fabricated from PP composites reinforced with woven flax fabrics, through cold NIF and SF processes performed both with and without a partial counter die [36]. Its main goal is to adopt a relatively simplified numerical modeling approach based on a thorough understanding of the material behavior, with particular attention to key phenomena such as elastic springback and damage evolution; this strategy aims to overcome the challenges associated with the complex characterization of this composite material, due to its unconventional stress transfer mechanism. The reliability of the proposed modeling approach was assessed through a comparison between numerical predictions and experimental observations, considering aspects such as the final geometry of the formed components, the trend of forming forces, and the occurrence of damage and failure mechanisms. The results demonstrated that FEM simulations, supported by an accurate understanding of material behavior, serve as an effective predictive tool for the design and optimization of forming processes involving these innovative lightweight composite structures.

## 2. Materials and Methods

This section summarizes the key characteristics of the composite laminates and the manufacturing procedures employed to produce the spherical caps. Furthermore, it outlines the FEM approach adopted to model and simulate the two forming processes.

### 2.1. Materials

The laminates simulated in this study, characterized by nominal thickness *t* = 2.2 mm, were manufactured using PP films (supplied by GDC S.r.l., Casorezzo, Italy) with a thickness of 0.5 mm and density of 0.92 g/cm^3^ and a woven flax fabric (supplied by FIDIA S.r.l.—Technical Global Services, Milano, Italy) not subjected to any previous chemical or surface treatment, with a density of 1.5 g/cm^3^ mass per unit area of 320 g/cm^2^ and tex number of 324 g/km. The main properties of the single unimpregnated yarns were: tensile strength of 512 MPa; tensile modulus of 21.4 GPa; elongation at break of 3.27% [42].

The laminates had the following stacking sequence: a five-layer symmetric layup; the first two and the last two layers were PP films, while the central layer was flax fabric. They were obtained by a compression molding process carried out at a pressure of 4 MPa at 200 °C, for a total time of 300 s; further details of the process can be found in [42], while the mechanical behavior of the manufactured laminates, with particular reference to their tensile properties, is reported in [36].

### 2.2. Forming Processes

**Figure 1:**
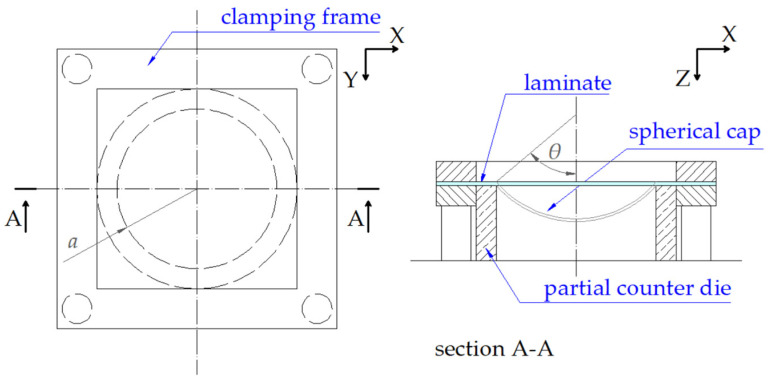
CAD model of the experimental setup and main geometrical characteristics of the spherical caps.

Spherical caps with a base radius *a* and a polar angle *θ* were produced through cold NIF and SF processes. The experimental activities were carried out using a high-speed four-axis vertical machining center (C.B. Ferrari S.r.l. Mornago, Italy), while the composite laminates were held in place by a clamping frame providing a square forming area of 100 × 100 mm^2^. To mitigate sheet-bending defects occurring near the base region of the caps, additional tests were performed using a partial counter die consisting of a hollow cylindrical support with internal and external diameters of 80 mm and 100 mm, respectively. Furthermore, all forming experiments were conducted under lubricated conditions to minimize friction-related defects and reduce the likelihood of material failure. A CAD model of the experimental setup, together with the main geometrical characteristics of the manufactured spherical caps, is presented in Figure 1.

Regarding the forming tools, the NIF experiments were carried out using a non-rotating stylus with a hemispherical tip having a diameter of 10 mm. The tool followed a helical trajectory at a speed *v_NIF_* = 1000 mm/min, with successive turns alternating between clockwise and counterclockwise directions. Further details of the toolpath strategy can be found in [36]. In the SF tests, hemispherical tools matching the dimensions of the target spherical caps were employed. Unlike the NIF process, the forming tool did not follow a complex trajectory but was driven along a single vertical path at a speed *v_SF_* = 60 mm/min.

### 2.3. FEM Simulations

FEM simulations were carried out to replicate the experimental forming tests using the commercial general-purpose software MSC Marc/Mentat 2025.2, which employs an implicit nonlinear solution scheme [43]. Five experimental configurations were simulated, labelled as NIF_0_, NIF_PD_, SF_0_, SF_PD_, and SF*_0_. For the details, see the table of abbreviations.

The numerical model was developed within the Mentat pre-processing environment and includes the composite sheet, the forming tools, and the partial counter die. The square laminate, with dimensions of 100 mm × 100 mm, was meshed using 7500 structural 3-D solid shell elements, corresponding to 50 elements along each side and three elements through the thickness. The forming tools and the partial counter die were modeled as rigid walls; in detail, the forming tools were modeled as spheres, while for the partial counter die only the surface in contact with the laminate was modeled.

The tool trajectory was defined by using the position control option for rigid contact bodies and prescribing controlled translational displacements along the X, Y, and Z axes through time–displacement tables, while zero displacements were assigned to the partial counter die. To reproduce the action of the clamping frame, all peripheral nodes of the sheet were constrained by imposing zero displacements in the X, Y, and Z directions. A friction coefficient of 0.1 was assumed for all contact interactions.

Modeling the material behavior represented the most challenging aspect of the numerical simulations. As reported in [36], tensile tests revealed failure occurring perpendicular to the yarns, with the flax fibers acting as reinforcement for the PP matrix in terms of both tensile strength (from 27.1 to 37.3 MPa) and elastic modulus (from 1.2 to 2.3 GPa). Stress transfer was primarily governed by mechanical interlocking, promoted by the relatively coarse woven architecture [44,45], rather than by fiber/matrix adhesion, which is inherently weak in this composite system because of the dissimilar chemical nature of flax fibers and the PP matrix, particularly in the absence of fiber surface treatments and coupling agents [46]. Nevertheless, the mechanical interlocking is not sufficiently strong to prevent the nearly linear plastic flow of the PP matrix up to the ultimate tensile strength.

Since the material does not exhibit the typical mechanical response of conventional composite laminates, it was modeled using an isotropic elastic–plastic model, while accounting for its distinctive characteristics. Specifically, an elastic modulus of 2.3 GPa was assigned, with yielding at a stress of 15 MPa. Between the yield stress and the ultimate tensile strength, a linear hardening law was adopted up to a strain of 0.6, consistent with the ultimate strain of PP and with the reported ability of the matrix to undergo relative sliding with respect to the fibers due to the limited mechanical interlocking. To account for failure perpendicular to the yarns, a damage model was introduced along the X and Y directions, with a damage initiation stress of 37.3 MPa and a residual stiffness factor of 0.5. Finally, considering that the fibers provide reinforcement under tensile loading but not under compression and that the elastic modulus of PP decreases with increasing plastic deformation [47], a variable elastic modulus model was adopted. In the plastic regime, the elastic modulus was progressively reduced to 10% of its initial value.

All simulations were performed using a constant time step of 0.2 s. The numerical results were subsequently post-processed and evaluated using the Mentat software environment.

## 3. Results and Discussion

**Figure 2:**
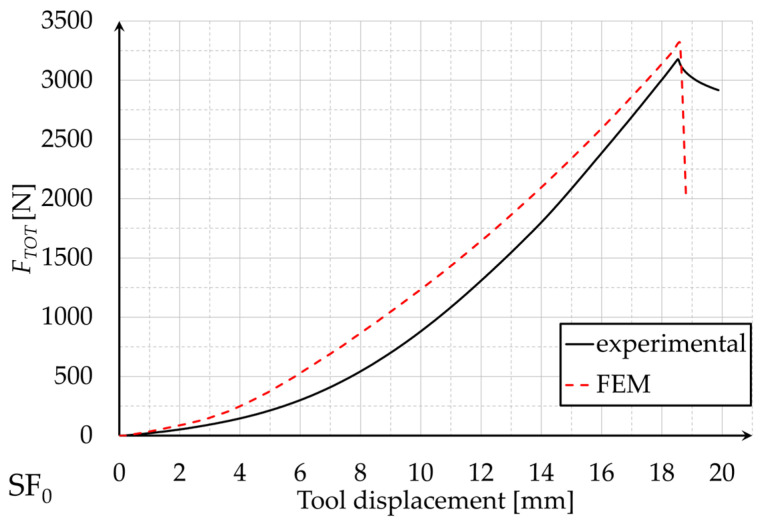
Numerical–experimental *F_TOT_* trends for the SF_0_ test.

**Figure 3:**
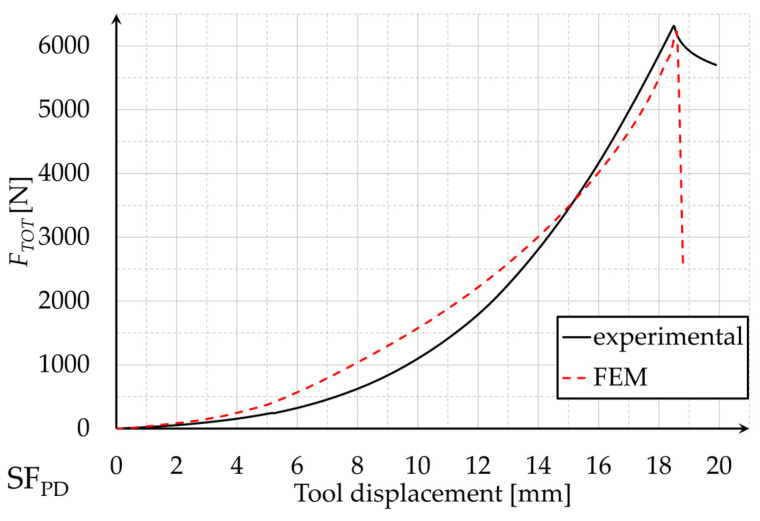
Numerical–experimental *F_TOT_* trends for the SF_PD_ test.

**Figure 4:**
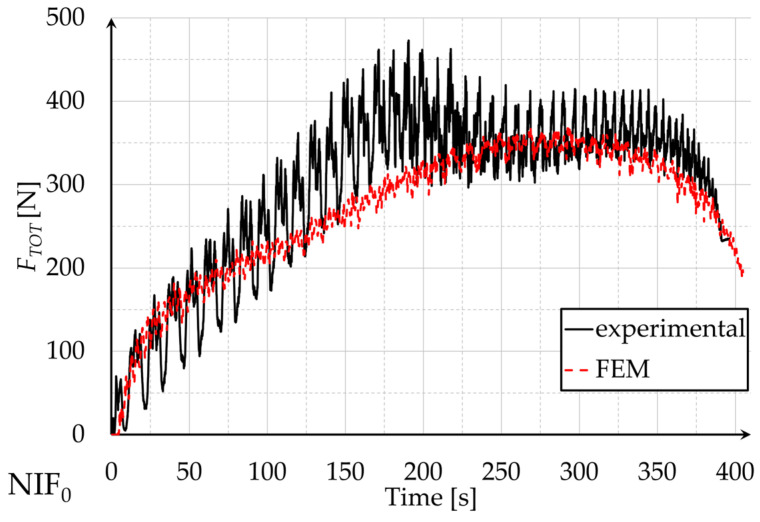
Numerical–experimental *F_TOT_* trends for the NIF_0_ test.

**Figure 5:**
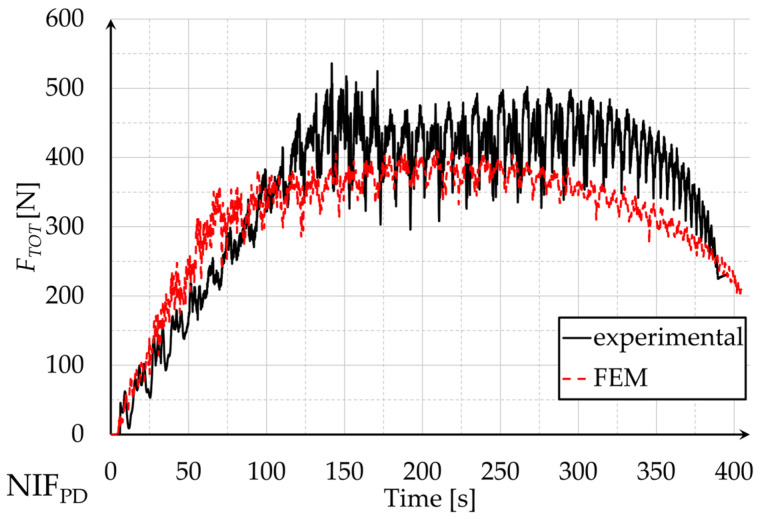
Numerical–experimental *F_TOT_* trends for the NIF_PD_ test.

Consistent with the experimental observations, the FEM simulations corresponding to the first four test configurations (SF_0_, SF_PD_, NIF_0_ and NIF_PD_) did not predict the occurrence of failure. This conclusion is supported by the corresponding forming force histories, which exhibit trends without any abrupt variations (except for the final stage of the processes) typically associated with damage initiation. A comparison between the numerical and experimental results in terms of total forming force (*F_TOT_*) is presented in Figure 2, Figure 3, Figure 4 and Figure 5. Note that different independent variables were adopted for the two forming processes: tool displacement was used for the SF tests, whereas process time was selected for the NIF tests.

**Figure 6:**
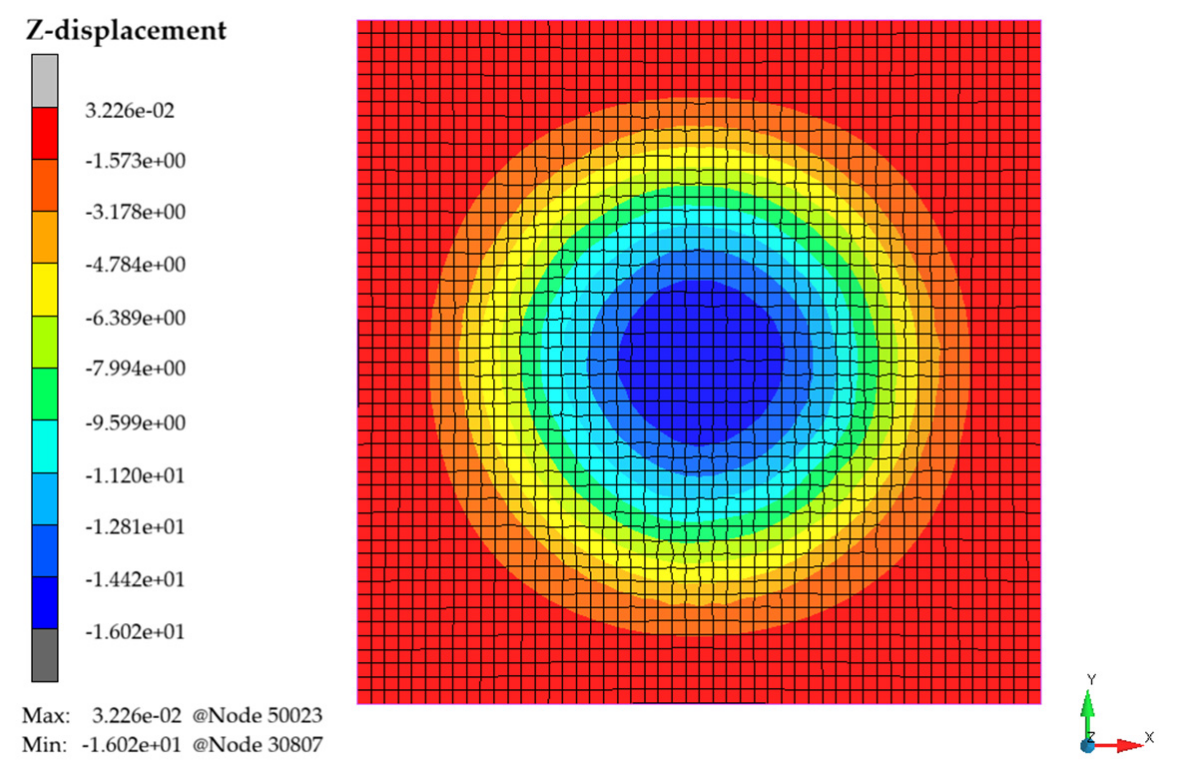
Z-displacement FEM plot for the NIF_0_ test.

The spherical caps produced by NIF did not exhibit any noticeable twisting. This can be attributed to the alternating toolpath strategy, whereby the twist generated during one turn is almost entirely compensated in the subsequent turn [36]. This observation is confirmed by the numerical simulations. For example, Figure 6 presents the Z-displacement contour bands plot in an XY view, obtained at the end of the NIF_0_ simulation. The absence of asymmetric deformation patterns provides clear evidence that no twisting occurred during the forming process, in agreement with the experimental observations.

**Figure 7:**
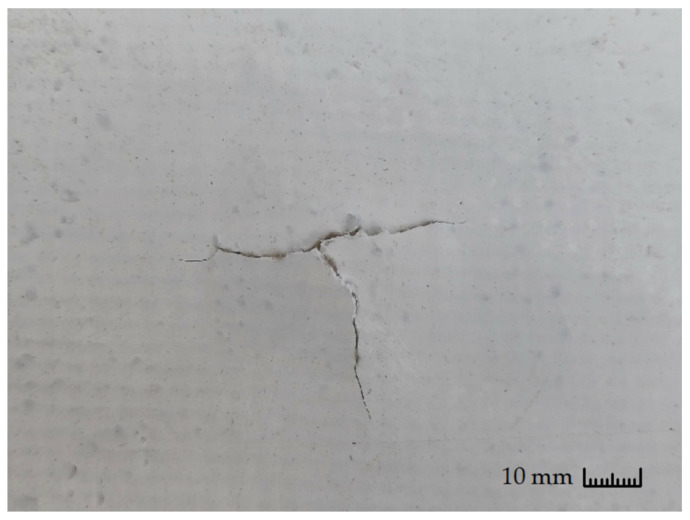
Laminate failures for the SF*_0_ test.

**Figure 8:**
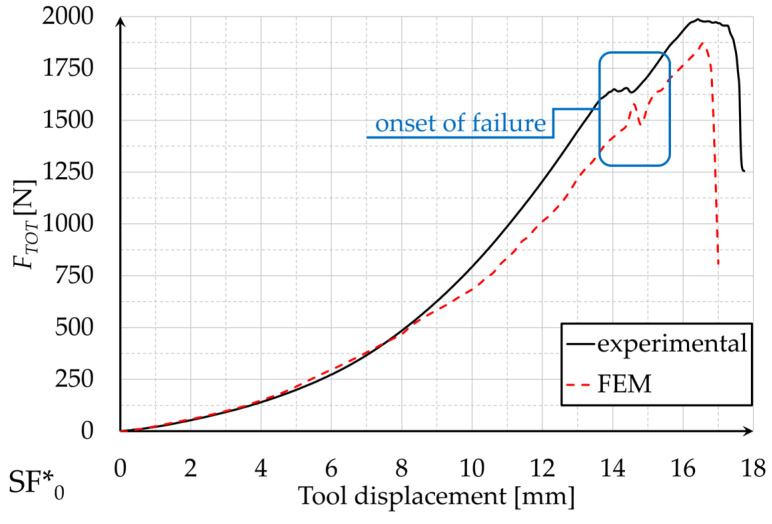
Numerical–experimental *F_TOT_* trends for the SF*_0_ test.

The SF*_0_ test ended with the occurrence of failure, characterized by cracking of the laminate perpendicular to the fibers’ direction (see Figure 7, showing the extrados of the laminate). This damage mechanism can be attributed to excessive loading of the polymer matrix following the fracture of the reinforcing fibers [48]. As shown in Figure 8, the onset of failure is clearly identified by the abrupt change in the force–displacement curve recorded during the experiment. Following failure, the force decreases but does not vanish because of the residual load-carrying capacity of the laminate. These results also demonstrate the effectiveness of incorporating a damage model into the FEM simulations. The numerical model successfully predicted the occurrence of failure. Specifically, the FEM results indicate the initiation of material damage at a tool displacement of 14.6 mm, in good agreement with the experimental observation (14.1 mm). At failure, the predicted and experimental forces are approximately 1580 and 1640 N, respectively.

Regarding the forming force trends, the FEM predictions show good overall agreement with the experimental measurements. This agreement is especially evident for the SF processes. Considering the characteristic force values at the end of the tests (i.e., the peak values of *F_TOT_* in Figure 2 and Figure 3, and the value of *F_TOT_* at failure in Figure 8), the maximum deviation does not exceed 4.5%. The observed discrepancies can be mainly attributed to the simplified material model adopted in the simulations, which does not fully capture the complex mechanical behavior of the composite laminates and inevitably limits the accuracy of the numerical response. As for the force peaks observed during the NIF processes, these features are not reproduced with the same magnitude in the numerical simulations. A more accurate representation could likely be achieved by employing a finer finite element mesh and smaller time steps because of the localized and incremental deformation mechanism typical of the ISF processes. However, such refinements would significantly increase the computational effort and simulation time, which are already considerable for NIF processes due to their inherently slow and incremental nature.

**Figure 9:**
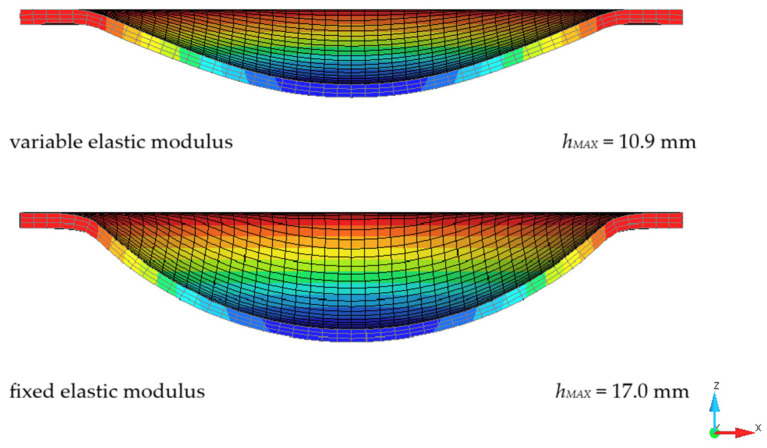
XY cross-section of SF_PD_ caps from FEM simulations with variable and fixed elastic modulus.

Another relevant aspect is elastic springback. To evaluate this phenomenon, the maximum height of the caps after tool removal at the end of the process (*h_MAX_*) was considered. Table 1 reports the experimental and FEM values of this feature for the tests completed without the occurrence of failure; note that, for these cases, the target height was 18.6 mm. As shown in Table 1, elastic springback is particularly pronounced in the SF tests due to the stretching stresses affecting the entire laminate, whereas the incremental deformation induced by the NIF tests mitigates this undesirable effect. Although the FEM simulations successfully reproduce the springback phenomenon, they do not accurately match the experimental values. These results indicate that the adoption of a variable elastic modulus provides an effective strategy for reproducing elastic springback. In this regard, Figure 9 compares the FEM simulations of the SF_PD_ tests performed using fixed and variable elastic modulus models. The figure clearly shows that the deviation from the target geometry is considerably greater when a fixed elastic modulus is adopted. As with the trend of the forces discussed above, improved agreement could be achieved through a more accurate characterization of the material behavior. Nevertheless, accurately characterizing the material remains a major challenge. For example, several aspects of the nonlinear unloading–reloading behavior, including the relationship between a plastic-strain-dependent chord modulus and the representation of the nonlinear stress–strain response, are still not fully understood [49].

## 4. Conclusions

This study presented a FEM framework for simulating the cold negative incremental forming and stretch forming of woven flax fiber-reinforced polypropylene composite laminates, both with and without a partial counter die. Owing to the unconventional behavior of the investigated material, a simplified isotropic elastic–plastic constitutive model, incorporating elastic springback and damage initiation, was adopted to achieve a satisfactory compromise between computational efficiency and predictive accuracy.

The numerical results showed good agreement with the experimental data. In particular, the proposed model successfully reproduced the force–displacement response, including the peak forming forces, the elastic recovery of the laminates after unloading, and the onset of material failure. These findings demonstrate that the proposed finite element framework provides a reliable predictive tool for the design and optimization of forming processes involving natural fiber-reinforced thermoplastic composites. For example, accurate prediction of the forming forces can support the proper sizing of the forming equipment, while reliable prediction of the final geometry and the onset of failure provide valuable information for assessing material formability and optimizing both the process parameters and the toolpath strategy.

Future work will focus on improving the material constitutive description through a more comprehensive experimental characterization of the unloading response, post-yield behavior, and damage evolution. Moreover, dedicated friction tests at the tool–sheet interface will be performed to calibrate the contact conditions more accurately, thereby further improving the robustness and applicability of the proposed framework.

## Figures and Tables

**Table 1:** Experimental and FEM values of *h_MAX_*.

Case	Experimental [mm]	FEM [mm]
SF_0_	5.2	8.0
SF_PD_	5.9	10.9
NIF_0_	15.3	15.6
NIF_PD_	15.9	15.8

## Data Availability

The original contributions presented in this study are included in the article. Further inquiries can be directed to the corresponding author.

## Abbreviations

The following abbreviations are used in this manuscript:

PP	Polypropylene
ISF	Incremental sheet forming
NIF	Negative incremental forming
CNC	Computerized numerical control
FEM	Finite element method
RVE	Representative volume element
*t*	Laminate thickness
*a*	Base radius of the spherical caps
*θ*	Polar angle of the spherical caps
CAD	Computer-aided design
*v_NIF_*	Speed of the tool for negative incremental forming processes
*v_SF_*	Speed of the tool for stretch-forming processes
NIF_0_	Negative incremental forming without partial counter die for spherical caps with base radius *a* = 40 mm and polar angle *θ* = 50°
NIF_PD_	Negative incremental forming with partial counter die for spherical caps with base radius *a* = 40 mm and polar angle *θ* = 50°
SF_0_	Stretch forming without partial counter die for spherical caps with base radius *a* = 40 mm and polar angle *θ* = 50°
SF_PD_	Stretch forming with partial counter die for spherical caps with base radius *a* = 40 mm and polar angle *θ* = 50°
SF*_0_	Stretch forming without partial counter die for spherical caps with base radius *a* = 20 mm and polar angle *θ* = 70°
*F_TOT_*	Module of the total forming force
*h_MAX_*	Maximum height of the caps
